# A practical biphasic contrast media injection protocol strongly enhances the aorta and pulmonary artery simultaneously using a single CT angiography scan

**DOI:** 10.1186/s12880-021-00691-4

**Published:** 2021-10-30

**Authors:** Cheng-Chih Hsieh, An-Bang Zeng, Chia-Hung Chen, Zong-Yi Jhou, Chih-Hsin Wang, Ya-Ling Yang, Feng-Chuan Hsieh, Jing-Kai Lin, Ju-Yen Yeh, Chun-Chao Huang

**Affiliations:** 1 Department of Radiology, MacKay Memorial Hospital, No.92, Sec.2, Zhongshan North Rd., Taipei, 10449 Taiwan; 2 Department of Medicine, MacKay Medical College, New Taipei City, Taiwan; 3 Mackay Junior College of Medicine, Nursing, and Management, Taipei, Taiwan

**Keywords:** Computed tomography angiography, Aortic dissection, Pulmonary embolism, Contrast media

## Abstract

**Background:**

Enhancement profiles of the pulmonary artery (PA) and aorta differ when using computed tomography (CT) angiography. Our aim was to determine the optimal CT protocol for a one-time CT scan that assesses both blood vessels.

**Methods:**

We prospectively enrolled 101 cases of CT angiography in patients with suspected pulmonary embolism or aortic dissection from our center between 2018 and 2020. We also retrospectively collected the data of 40 patients who underwent traditional two-time CT scans between 2015 and 2018. Patients were divided into four groups: test bolus (TB) I, TB II, bolus-tracking (BT) I, and BT II. The enhancement of the PA and aorta, and the radiation doses used in the four groups were collected. Those who underwent two-time scans were classified into the traditional PA or aorta scan groups. Data were compared between the BT and traditional groups.

**Results:**

The aortic enhancement was highest in BT II (294.78 ± 64.48 HU) followed BT I (285.18 ± 64.99 HU), TB II (186.58 ± 57.53 HU), and TB I (173.62 ± 69.70 HU). The radiation dose used was lowest in BT I (11.85 ± 5.55 mSv) and BT II (9.07 ± 3.44 mSv) compared with that used in the traditional groups (20.07 ± 7.78 mSv) and accounted for half of the traditional group (45.17–59.02%). The aortic enhancement was also highest in BT II (294.78 ± 64.48 HU) followed by BT I (285.18 ± 64.99 HU) when compared with that in the traditional aorta scan group (234.95 ± 94.18 HU).

**Conclusion:**

Our CT protocol with a BT technique allows for a lower radiation dose and better image quality of the PA and aorta than those obtained using traditional CT scans.

*Trial registration*: NCT04832633, retrospectively registered in April 2021 to the clinical trial registry.

## Background

Acute chest pain is a common chief complaint in the emergency department [1]. Pulmonary embolism (PE) and aortic dissection (AD), life-threatening diseases with high mortality rates, share similar symptoms that include acute chest pain [2, 3]. In daily practice, clinical physicians battle to differentiate PE and AD because of their similar presentations. To improve clinical management and avoid catastrophic consequences, an accurate diagnosis of PE and AD should be established.

Computed tomography (CT) is required for the precise evaluation of both PE and AD, and CT angiography (CTA) is highly sensitive for diagnosing PE and AD [4]. However, the peak enhancement times for the pulmonary artery (PA) and aorta are different, which raises concerns on whether obtaining optimal pulmonary angiograms and aortograms simultaneously is feasible [5–7]. Traditional two-time CT scans for pulmonary angiography and aortography elevate the risk of radiation exposure, contrast-induced nephropathy, and allergies [8, 9]. Theoretically, the radiation exposure in traditional two-time CT scans is higher than that in a one-time CT scan. Our protocol is related to triple-rule-out CT angiography, but there are several differences between them [10, 11]. Triple-rule-out CT angiography is designed for acute coronary syndrome. Therefore, demonstration of the coronary arteries is essential, there are many specific requirements for this technique, including a high injection rate (at least 5 mL/s), ECG-gating, and administrationof beta-blockers and sublingual nitroglycerin, and there is a limited scan range from the aortic arch to the base of the heart. Although this technique can detect the aorta and pulmonary arteries simultaneously, it fails to cover the abdominal aorta and some distal branches of the PA. In contrast, our scan protocol was designed for AD and PE based on initial clinical evaluation. Therefore, our protocol has no specific preparation requirements for imaging coronary arteries and can have a scan range that covers the entire aorta and the pulmonary arteries. No study thus far has presented a one-time CT scan protocol for diagnosing PE and AD simultaneously.

In this study, we designed a one-time CT scan protocol with different contrast medium injection methods to decrease radiation exposure but preserve image quality for diagnosing PE and AD simultaneously.

## Methods

### Patients

All cases of suspected PE or AD referred for CTA in our hospital between March 2018 and February 2020 were reviewed. Patients with a known allergic history, poor renal function (creatinine level > 1.5 mg/dL), pregnant women, or those aged less than 20 years were excluded from the study. Finally, we enrolled and analyzed 101 CTA cases. Data collected included age, sex, blood pressure, body mass index (BMI), Hounsfield unit (HU) at bifurcation of PA, HU at the descending aorta with the same level of PA bifurcation, time to peak enhancement, and radiation dose. This study was approved by the Institutional Review Board of our hospital. Written informed consent was obtained from all patients. Retrospectively, 40 cases of suspected PE or AD with traditional two-time CT scans were compared with the experimental groups.

### Experimental design of contrast media injection

In the prediagnostic axial CT images, the region of interest (ROI) was set at the level of the PA bifurcation using a 10 mL test bolus (TB) contrast media injection at a rate of 3 mL/s. The CT densities were collected and time-enhancement data were demonstrated. The contrast media was injected from the peripheral veins with systemic circulation to the PA. The interval from contrast injection to the peak enhancement of the PA was defined as P seconds. After pulmonary circulation, the contrast medium contracted into the aorta. The interval from contrast injection to the peak enhancement of the aorta was defined as A seconds.

We designed a biphasic contrast media injection protocol that included a low rate (2 mL/s) first phase injection and a high rate (3 mL/s) second phase injection followed by a normal saline injection. The first phase injection was maintained for (A-P) seconds, and the second phase injection was maintained until the rest of the contrast media was completely injected. Theoretically, when the start time of the scan is at A seconds, the first phase contrast media will significantly enhance the aorta, although the peak time and enhancement will not be optimal because the time intervals are calculated based on the high rate (3 mL/s) injection. Conversely, the second phase contrast media will maximally enhance the PA at the same time because it has been injected for P seconds [A−(A−P) = P].

**Fig. 1:**
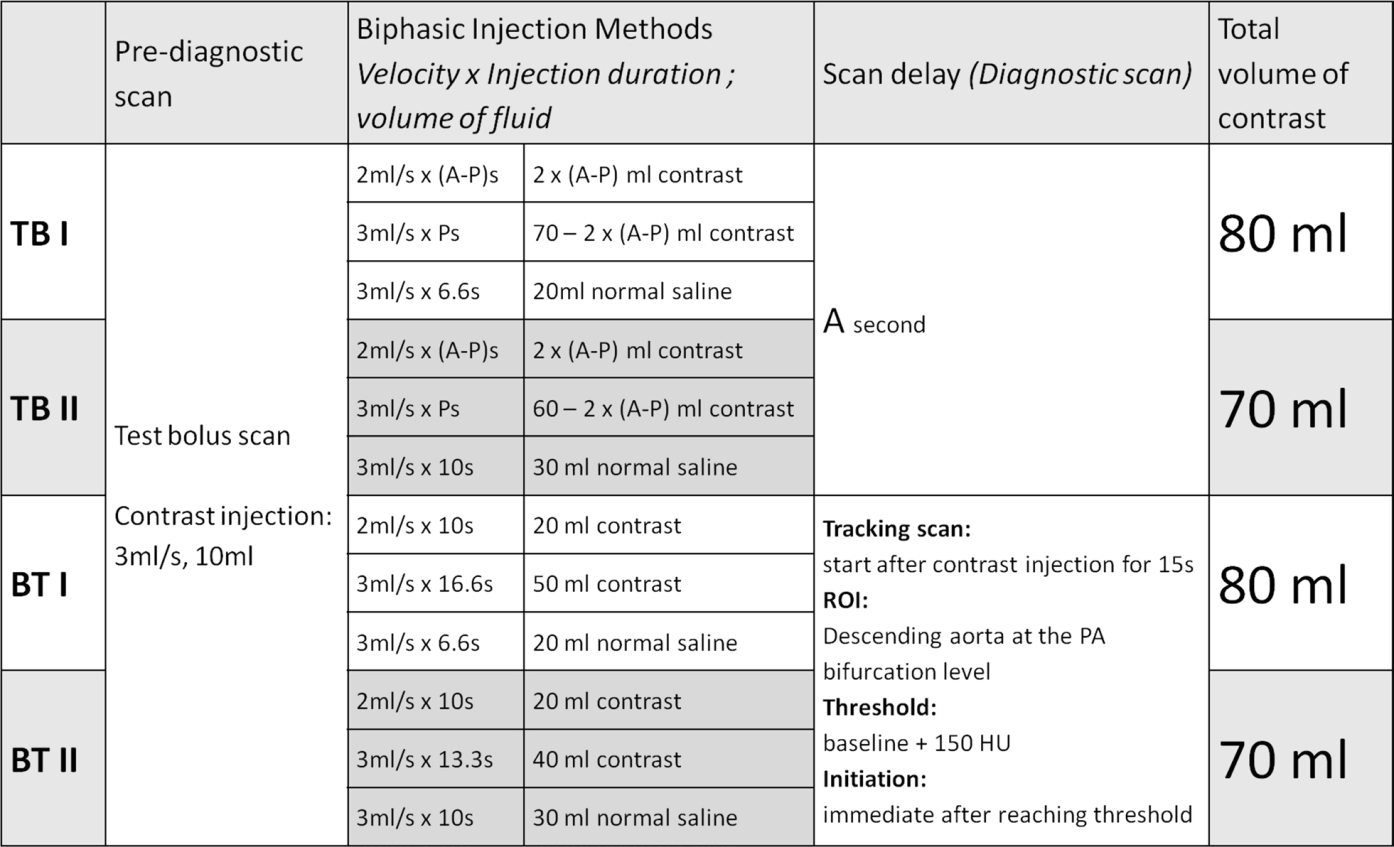
The details of the contrast medium injections and CT scan protocols of the four experimental groups. TB: test bolus; BT: bolus-tracking; A: peak enhancement time of the aorta on prediagnostic scan; P: peak enhancement time of the pulmonary artery on prediagnostic scan; PA: pulmonary artery; ROI: region of interest

We designed four groups: two that used the TB method and two that used the bolus-tracking (BT) method. The two TB groups had a total amount of contrast medium of either 80 mL or 70 mL. The scan start time was fixed at A seconds. Based on the results of the initial 22 cases in the TB groups, the mean A-P interval was 9.5 s. Therefore, we assumed that the A-P interval in most cases was approximately 10 s, and designed the two BT groups with a fixed 10-s first phase contrast media injection time. The start time of the scan was initiated when the descending aorta at the PA bifurcation achieved a baseline density of 150 HU. The two BT groups also had a total amount of contrast medium of either 80 mL or 70 mL. Figure 1 provides a summary of the four groups.

### Computed tomography scan technique and administration of contrast medium

CT was performed using a 320-MDCT scanner (Canon Aquilion PRIME CT scanner, Toshiba Medical Systems, Otawara, Japan). The scan parameters were as follows: 5 mm section thickness, 100 kVp, automatic exposure control of current (mA), and 0.5-s rotation time. All patients were given a breath-hold instruction for the standard diagnostic CT in the craniocaudal direction.

**Fig. 2:**
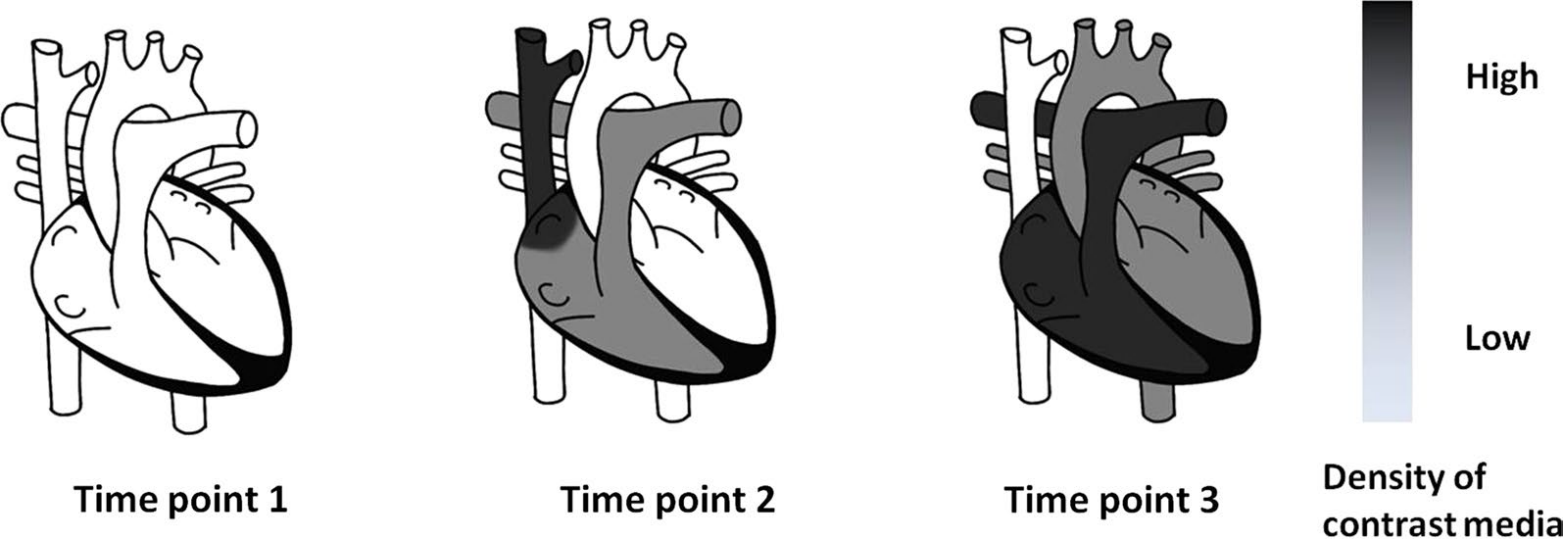
Black: high density of contrast medium. White: low density of contrast medium. Time point 1 is before contrast medium injections; therefore, all the vascular structures are white. Time point 2 is about the midway of the two contrast injection phases and the first slowly injected contrast media, shown in gray color, arrive the pulmonary artery and at the same time, the second fast injected contrast media, shown in black color, arrive the superior vena cava. There is very low contrast media in the aorta at this time point. Time point 3 is the start point of the diagnostic scan. At this time point, the first slowly injected contrast media, shown in gray color, arrive the aorta and at the same time, the second fast injected contrast media, shown in black color, arrive the pulmonary artery

All patients were administered with contrast medium with 300 mg iodine (I)/mL Omnipaque using a dual-power injector (MEDRAD Stellant CT injector). In all cases, right upper limb antecubital venous access was achieved using an 18- or 20-gauge venous catheter. The predicted enhancement pattern during the contrast medium injections using the biphasic contrast injection protocol followed by a saline flush is shown in Fig. 2.

### Radiation dose and statistical analysis

The dose length product (DLP) was recorded in all cases. Our study applied a conversion factor of 0.014 mSV/(mGy × cm) based on the region of the chest and abdomen [12]. One-way analysis of variance (ANOVA) was used to compare the data of different groups. If the test of homogeneity of variances was not significant, the Scheffe method was used for post hoc analysis. Otherwise, the Games-Howell method was used for post hoc analysis. Statistical significance was set at *p* ≤ 0.05. All statistical analyses were performed using SPSS software (SPSS version 25.0, IBM Corp.).

## Results

### Comparison between the four experimental groups

In total, 21 patients were included in the TB I group, 17 in the TB II group, 37 in the BT I group, and 26 in the BT II group. ANOVA showed significant differences in sex between the four groups: TB I (men: 15/21 = 71%), TB II (men: 9/17 = 53%), BT I (men: 26/37 = 70%), and BT II (men: 10/26 = 38%) (*p* = 0.043). However, post hoc analysis using the Scheffe method showed no significant differences in sex between the groups. A similar result was noted for radiation dose in the ANOVA: TB I (8.49 ± 4.18 mSv), TB II (8.65 ± 5.33 mSv), BT I (11.85 ± 5.55 mSv), and BT II (9.07 ± 3.44 mSv) (*p* = 0.023). But there was no significant difference in radiation dose between the groups in post hoc analysis using the Scheffe method. ANOVA showed that the CT density of the aorta was significant (*p* < 0.001). Post hoc analysis using the Scheffe method showed that the density was highest in BT II (294.78 ± 64.48 HU) followed by BT I (285.18 ± 64.99 HU), TB II (186.58 ± 57.53 HU), and TB I (173.62 ± 69.70 HU) (BT vs TB, *p* < 0.001). There was no significant difference between BT I and BT II or between TB I and TB II. Furthermore, ANOVA showed no significant differences in age, heart rate, systolic and diastolic blood pressure, BMI, or CT density of the PA between the four groups (Table 1). The two BT groups presented the best image quality in terms of enhancement of the aorta with similar enhancement of the PA and the lowest radiation dose.

**Table 1:** Comparison between the four experimental groups

Group	TB I (N = 21)	TB II (N = 17)	BT I (N = 37)	BT II (N = 26)	*P*-value
Sex	Men: 15 (71%)	Men: 9 (53%)	Men: 26 (70%)	Men: 10 (38%)	**0.043**^**+**^
Age (years)	66.43 ± 17.38	66.94 ± 16.37	57.49 ± 13.84	62.85 ± 15.22	0.089
Heart rate (beats/min)	82.43 ± 21.86	80.76 ± 17.84	78.92 ± 17.11	74.81 ± 14.50	0.495
Systolic BP (mmHg)	128.57 ± 25.39	137.88 ± 24.79	143.86 ± 28.79	144.85 ± 26.89	0.148
Diastolic BP (mmHg)	80.67 ± 14.83	81.94 ± 15.69	88.68 ± 19.70	85.46 ± 9.36	0.248
BMI (kg/m^2^)	23.61 ± 2.84	24.05 ± 5.36	25.38 ± 3.79	24.59 ± 3.22	0.345
PA density (HU)	358.26 ± 104.29	295.89 ± 84.39	324.03 ± 99.90	285.19 ± 108.70	0.078
Aorta density (HU)	173.62 ± 69.70	186.58 ± 57.53	285.18 ± 64.99	294.78 ± 64.48	** < 0.001**
Radiation dose (mSv)	8.49 ± 4.18	8.65 ± 5.33	11.85 ± 5.55	9.07 ± 3.44	**0.023**^**+**^

Bold text indicating a statistically significant difference with a *P*-value less than 0.05

N: number; TB: test bolus; BT: bolus-tracking; BP: blood pressure; BMI: body mass index; PA: pulmonary artery

^**+**^ No statistically different in post hoc analysis using Scheffe method

### Comparison between the BT and traditional groups

Forty patients who underwent two-time CT scans were included in the traditional group. There were 21 men (21/40 = 53%) in this group. The average age was 73.35 ± 18.22 years, and the average radiation dose was 20.07 ± 7.78 mSv. The test of homogeneity of variances between the traditional and two BT groups showed significant differences in sex (*p* = 0.004) and radiation dose (*p* = 0.029). Post hoc analysis using the Game-Howell method showed no significant difference in sex but the radiation dose was significantly higher in the traditional than BT groups. The radiation dose of BT I was approximately 59.02% of that in the traditional group and that of BT II was approximately 45.17%. The test of homogeneity of variances between the traditional and two BT groups showed no significant differences in age. ANOVA using the Scheffe method showed that the average age in the traditional group was significantly higher than that in BT I (*p* = 0.001), but there was no significant difference between the traditional group and BT II.

To analyze the image quality, we separated the traditional two-time CT scans into the PA and aorta scans. The average CT densities of the PA were 302.20 ± 86.37 HU in the PA scan and 195.33 ± 71.44 HU in the aorta scan. The average CT densities of the aorta were 165.68 ± 80.95 HU in the PA scan and 234.95 ± 94.18 HU in the aorta scan. The tests of homogeneity of variances were significant in the CT density of the PA (*p* = 0.014) and that of the aorta (*p* = 0.005) between the two scan and BT groups. Post hoc analysis of PA enhancement using the Game-Howell method showed that the enhancement was similar between the two BT and PA scan groups but the least in the aorta scan group. Post hoc analysis of aortic enhancement using the Game-Howell method showed that the enhancement was similar between and highest in the two BT groups compared with the aorta and PA scan groups (Table 2).

**Table 2:** Comparison between the BT and traditional groups

Group	Traditional (Aorta scan)	Traditional (PA scan)	BT I	BT II	Test of homogeneity of variances	ANOVA *P*-value
Sex	M:21(53%)		M:26(70%)	M:10(38%)	**0.004**	
Age (years)	73.35 ± 18.22		57.49 ± 13.84	62.85 ± 15.22	0.311	** < 0.001**
PA density (HU)	195.33 ± 71.44	302.20 ± 86.37	324.03 ± 99.90	285.19 ± 108.70	**0.014**	
Aorta density (HU)	234.95 ± 94.18	165.68 ± 80.95	285.18 ± 64.99	294.78 ± 64.48	**0.005**	
Radiation dose (mSv)	20.07 ± 7.78		11.85 ± 5.55	9.07 ± 3.44	**0.029**	

Group	Traditional versus BT I	Traditional versus BT II	BT I versus BT II			
*Post hoc analysis using the Games-Howell method (p-value)*						
Sex	0.380	0.682	0.061			
Radiation dose (mSv)	** < 0.001**	** < 0.001**	0.079			

Group	T_Ao versus T_PA	T_Ao versus BT I	T_Ao versus BT II	T_PA versus BT I	T_PA versus BT II	BT I versus BT II
PA density (HU)	** < 0.001**	** < 0.001**	**0.003**	0.737	0.907	0.479
Aorta density(HU)	**0.004**	**0.038**	**0.017**	** < 0.001**	** < 0.001**	0.938

Bold text indicating a statistically significant difference with a *P*-value less than 0.05

PA: pulmonary artery; BT: bolus-tracking; ANOVA: Analysis of variance; T_Ao: Traditional(Aorta scan); T_PA: Traditional(PA scan)

## Discussion

In this study, the BT groups showed better imaging results than did the TB groups to achieve high enhancement of the PA and aorta in a one-time CT scan. Furthermore, compared with the traditional two-time scan groups, the BT groups showed the best enhancement of the PA and aorta, and the lowest radiation dose.

### Image quality

In this study, we chose to use HU values as the objective parameter to evaluate image quality. Image quality can be assessed using objective physical parameters or the human observer approach, but the interpretation of the image quality by different physicians can vary greatly [13, 14]. Methods such as the signal-to-noise ratio and maximum intensity projection are used to objectively evaluate image quality [15]. The HU value was used as an objective criterion in our experimental design. The higher the HU value, the better the image quality. The comparison between the four experimental groups in this study showed similar enhancement of the PA, but the enhancement of the aorta was higher in the BT than TB groups. Therefore, the two BT groups have the best image quality. The enhancement of the PA in the two BT groups was similar to that in the traditional PA scan group but higher than that in the traditional aorta scan group. Compared with the traditional aorta and PA scan groups, the two BT groups had the highest enhancement of the aorta. Therefore, the two BT groups presented better image quality and overall performance than did the traditional scan groups.

### Time to peak enhancement

Although optimal attenuation for the great vessels varies, the required enhancement of the PA and aorta is approximately 180–300 HU [8, 16, 17]. The time to peak enhancement of the PA and aorta is approximately 6–8 s and 15–18 s, respectively [5, 8, 18]. The mean enhancement of the PA was 285.19–358.26 HU in the four experimental groups and of the aorta was 285.18–294.78 HU in the two BT groups, thus fulfilling the requirements. However, the mean enhancement of the aorta was only 173.62–186.58 HU in the two TB groups, which is considered unsatisfactory but might still be useful in clinical settings because the aorta is a large vessel. In this study, the mean P and A seconds were 10.87 s and 19.72 s, respectively, in the final experimental groups. The data we collected seem to be in accordance with the published literature but are slightly delayed [19]. There are several factors that affect the P and A time, such as the amount and rate of contrast media injection and individual hemodynamic status. The slight delay in our study could be due to different evaluation methods or study participants. Furthermore, the aim of this study was to find a single CT scan protocol with simultaneous optimal enhancement of the PA and aorta. Thus, the most important parameter is the interval between A and P time, which is approximately 9–10 s in the literature [19], which is comparable with our results.

To the best of our knowledge, the ongoing time-enhancement equation is based on Bae's study: scan delay (TSD, time of arrival + diagnostic delay) is considered as the scan starting time. TDELAY = time to peak contrast enhancement (TPEAK) − (1/2) × TSD. The TPEAK for CT pulmonary angiography is determined by the injection duration (TID) and contrast material arrival time (TARR): TPEAK = TID + TARR − 5 [8, 20]. The time to peak enhancement has many influencing factors, such as injection duration, scan duration, and individual hemodynamic status. Moreover, the diagnostic delay may vary for different CT scanners. Multiple factors complicate the use of these formulas for clinical application [8]. In particular, under multidetector CT, compared with the traditional scanning time, the scanning time is shorter. The time to peak enhancement requires a precise calculation. Our study is based on a rough hemodynamic circulation time calculation, but more complex protocols to achieve maximum enhancement of the PA and aorta can be established because of the many parameters affecting the scan.

### Test bolus and bolus-tracking

Both BT and TB apply to dynamic studies that require precise time for a specific organ [8]. There is not much difference between the two methods in image presentation, but the BT method is often preferred because of its efficiency and practicality [21–23]. In our study, the two BT groups obtained better images with simultaneous high enhancement of the PA and aorta than did the two TB groups. The TB design was based on a prediagnostic TB scan, and the BT design used a density threshold during the full contrast injection period. There are several key differences between these two designs. First, only 10 mL of contrast medium was used for the prediagnostic TB scan but 60–70 mL for the full contrast injection. The enhancement pattern might differ depending on the amount of contrast medium. Second, the full contrast injection in either BT or TB was biphasic instead of fixed at 3 mL/s, thus the enhancement pattern will be different from that in the prediagnostic TB scan. Although the TB design showed unsatisfactory results in our study, this method has the potential to identify hemodynamic status (A and P times) before the diagnostic scan. If the A–P time interval greatly exceeds the standard 9–10 s, the performance of the BT design will be affected [24]. Thus, although the BT design was easier and resulted in better image quality than the TB design, it might still be useful to perform a prediagnostic TB scan. Diagnostic images should be obtained using the BT method if the prediagnostic TB scan shows an A–P time interval of approximately 9–10 s. In contrast, the TB method is suggested to obtain diagnostic images, but further studies are required to confirm this suggestion.

### Biphasic injection

The biphasic injection method was divided into two phases with different velocities to improve earlier monophasic injections [25]. Monophasic injections show a Gaussian curve in time to enhance data in which the peak plateau cannot be maintained for a long period [8]. The biphasic injection method can solve this problem [26]. The two-phase injection reaches the peak of two phases (hump pattern), so that the middle of the time can reach a peak plateau for a period of time [8]. Martí-Bonmatí et al. showed no difference in velocities between the low–high and high-low biphasic injections [25].

Our experimental groups used biphasic injections with adjustments. The first phase used a low flow rate to ensure that the contrast bolus stayed at the aorta before the scan, and the second phase used a high flow rate to ensure sufficient enhancement of the PA. As expected, our design provided sufficient enhancement of the PA and aorta simultaneously. The enhancement of the PA was similar between the TB and BT groups, but the enhancement of the aorta was weaker in the TB than BT groups. In the PA images, there were no differences between the BT and traditional groups. In the aorta images, the BT groups achieved better enhancement than did the traditional groups.

### Saline flush

The clinical application of a saline flush has many benefits. The purpose of a saline flush is to reduce the intravascular contrast medium and reduce unnecessary viscous obstruction of the peripheral vessels. In a chest CT scan, a saline flush can reduce the influence of streak artifacts on the SVC and brachiocephalic veins, and decrease contrast-induced nephrotoxicity. In addition, a saline flush can strengthen enhancement, increasing peak enhancement and prolonging the time to peak enhancement (average, 3–10 s) [8, 27]. A previous study assessed the impact of varying amounts of saline on time to peak enhancement [27]. TB II and BT II had a high HU, in which less contrast medium with a higher volume of saline was used. However, our results do not support our claims among the four groups. We administered two different volumes (20 mL and 30 mL) of contrast medium at the same velocity (3 mL/s) in the four groups, but the enhancement within the BT and TB groups did not differ. This might be because of the small difference in the amount of saline used.

### Limitation

Our study has several limitations. First, it is difficult to objectively quantify image quality depending on individual variance. Second, the parameters of traditional groups cannot be achieved the same as experimental groups. These limitations limit the generalizability of the results to all populations and require further study for clinical application.

## Conclusion

We designed a modified biphasic injection method for a one-time CT scan. The PA images of the experimental groups showed equivalent image quality to that of the traditional groups. The image quality of the aorta in the experimental groups was better than that in the traditional groups. Moreover, the radiation dose of the experimental groups was significantly lower than that of the traditional groups (approximately half).

In summary, a patient suspected of having both PE and AD can be diagnosed with high enhancement of the PA and aorta within a single diagnostic CT scan using our CT protocol, especially the BT design.

## Data Availability

The datasets generated or analyzed in the current study are not publicly available because of patient privacy protection, but are available from the corresponding author upon reasonable request.

## Abbreviations

PE: Pulmonary embolism
AD: Aortic dissection
CT: Computed tomography
CTA: CT angiography
PA: Pulmonary artery
HU: Hounsfield unit
ROI: Region of interest
TB: Test bolus
BT: Bolus-tracking
